# A Smartphone App to Reduce Sugar-Sweetened Beverage Consumption Among Young Adults in Australian Remote Indigenous Communities: Design, Formative Evaluation and User-Testing

**DOI:** 10.2196/mhealth.8651

**Published:** 2017-12-12

**Authors:** Emma Tonkin, Lauren Jeffs, Thomas Philip Wycherley, Carol Maher, Ross Smith, Jonathon Hart, Beau Cubillo, Julie Brimblecombe

**Affiliations:** ^1^ Nutrition Program Wellbeing and Preventable Chronic Disease Menzies School of Health Research Casuarina, Northern Territory Australia; ^2^ Centre for Population Health Research School of Health Sciences University of South Australia Adelaide, South Australia Australia; ^3^ Wearable Computer Laboratory School of Information Technology and Mathematical Sciences University of South Australia Mawson Lakes, South Australia Australia; ^4^ Nutrition and Dietetics College of Nursing and Health Sciences Flinders University Bedford Park, South Australia Australia

**Keywords:** behavior, diet, health promotion, Indigenous, Telemedicine, mobile applications, public health

## Abstract

**Background:**

The disproportionate burden of noncommunicable disease among Indigenous Australians living in remote Indigenous communities (RICs) is a complex and persistent problem. Smartphones are increasingly being used by young Indigenous adults and therefore represent a promising method to engage them in programs seeking to improve nutritional intake.

**Objective:**

This study aimed to consult RIC members to inform the content of a smartphone app that can be used to monitor and reduce sugar-sweetened beverage intake in RICs.

**Methods:**

The study was conducted in two phases. The formative phase involved a simulated grocery selection activity with think aloud (“think aloud shop”), a semistructured interview, a questionnaire outlining current smartphone and app use, and a paper prototyping activity. A preliminary end-user testing phase involved a think aloud prototype test and a semistructured interview regarding user satisfaction. Convenience sampling was used to recruit 20 18- to 35-year-old smartphone users for each phase from two RICs in the Northern Territory, Australia. Thematic analysis of transcribed audio recordings was used to identify determinants of food choice from the think aloud shop; themes related to the Theory of Planned Behavior (TPB) from the eating behaviors interview; and usability, comprehension, and satisfaction with the app from the preliminary end-user testing.

**Results:**

Smartphone use in RICs is currently different to that found in urban environments; in particular, extremely low use of Facebook, restricted variety of phone types, and limited Internet access. Findings regarding promoting app engagement indicate that utilizing an opt-in approach to social features such as leader boards and team challenges is essential. The inclusion of games was also shown to be important for satisfaction, as were the use of audio features, contextually embedded dissemination, and streamlined app design for comprehension in this target group.

**Conclusions:**

This research provides critical insights and concrete recommendations for the development of lifestyle improvement apps targeted toward disadvantaged young adults in nonurban settings, specifically RICs. It serves as a framework for future app development projects using a consultative user-centered design approach, supporting calls for the increased use of this strategy in app development.

## Introduction

Effective strategies are needed to facilitate long-term weight control and improve health in Indigenous Australians living in remote communities, particularly in the young adult population who are still forming lifelong habits and often shaping those of their children. Smartphone apps are rapidly emerging as a tool to assist delivery and uptake of behavior change programs [1,2], with emerging evidence of modest efficacy in improving health-related behaviors in adolescent and young adult populations [3,4]. Smartphones and apps are increasingly being used by young Indigenous adults and therefore represent a promising method to engage them in programs seeking to improve nutritional intake [5]. The research reported in this paper intended to develop and test with Indigenous young adults living in remote communities the acceptability of an app designed with the aim of improving attitudes, self-efficacy, and intention to reduce sugar-sweetened beverage (SSB) intake.

Indigenous Australians, especially those who live in remote Indigenous communities (RICs), experience substantially worse health outcomes compared with other Australians [6]. Sixty-six percent of all Indigenous Australians (and 55% of those aged between 18 and 24 years) are overweight or obese [7]. This contributes to a disproportionately large burden of noncommunicable disease in this population, with 8.6% of Indigenous Australians living with diabetes; 3.9% with heart, stroke, and vascular diseases; and 1.8% with kidney disease [7]. Late adolescence and early adulthood (18-24 years) are important life stages for targeting interventions to prevent obesity and cardiometabolic disease in remote Indigenous Australians [8]. Additionally, as this age bracket is synonymous with preconception and parenthood for many young Indigenous adults, improving dietary intake at this point can benefit multiple generations [9].

Indigenous Australians living in RICs typically consume a nutritionally very poor diet that underpins the high rates of overweight and obesity. A key contributor to poor diet quality is the alarmingly high intake of SSBs; SSBs account for 10% of calories and 25% of food expenditure in RICs, with relatively higher intakes reported for young adults [10]. Several meta-analyses have shown that consumption of SSBs is independently associated with excess calorie intake, weight gain, and an increased risk of type 2 diabetes and cardiovascular disease [11,12]. Identifying effective strategies to reduce SSB intake, therefore, has the potential to make a substantial impact on dietary intake and weight status in this young adult population.

Apps and social media are rapidly evolving as tools to deliver targeted nutrition interventions at an individual level, as they can provide critical information and motivation for behavior change [1,13-15]. Access and use of smartphone technology, particularly by young adults, is rapidly growing with mobile network coverage now available in many RICs [5]. These technologies provide a cost-effective way to increase user interaction, provide peer-to-peer support, and widen access to health interventions [16] with the potential to modify behaviors [5]. Recent reviews of the evidence for the use of apps in improving health-related behaviors in adolescents have demonstrated modest improvements [3,4], suggesting apps to be a promising mode of delivery of health intervention for this typically difficult-to-reach population group. Systematic reviews of studies that have used apps to deliver diet and/or exercise interventions for weight loss have also found consistent evidence that digital technology–based/assisted interventions provide a weight loss advantage [17,18]. Although there are a multitude of apps targeting nutrition improvement available in the marketplace, many do not incorporate behavioral theory, and fewer still specifically target SSB consumption or disadvantaged populations [13,14].

The purpose of this study was to consult RIC members to inform the development of a smartphone app that can be used to monitor and reduce SSB intake in RICs. The aims of this research are threefold: to provide critical information for the future development of an app targeting SSB consumption in RICs; to afford insight into eating behaviors and smartphone use in the young adult RIC population which can be applied in many nutrition improvement and app development projects with disadvantaged, nonurban groups; and finally to contribute learnings about the application of best practice app development processes in an RIC context which can be broadly applied to disadvantaged and nonurban target populations internationally.

## Methods

### Preliminary Work

Prior to conducting this study, a scoping review [2] was performed to identify best practice methods for app development and end-user testing. The scoping review identified numerous app features that were successfully used to promote app engagement in adolescents, young adults and disadvantaged communities that were applicable to this study. Additionally, it highlighted key technical design elements important to consider during app development, such as app functionality without Internet connection. The research protocols for this study were subsequently designed based on the scoping review recommendations that were tailored to enable their application in the unique RIC setting.

This study was approved by the “Human Research Ethics Committee of Northern Territory Department of Health and Menzies School of Research” (HREC 20162659). All participants provided written, informed consent before participation.

### Study Design

The study was conducted in 2 phases. The structure is outlined below, and the methodological details of each component are further elaborated in the “data collection methods” section.

#### Phase 1: Formative Research

The formative research phase aimed to inform preliminary app features, content, and structure. It included the following 3 components: (1) a simulated grocery-selection activity with think aloud (henceforth “think aloud” shop); (2) a semistructured interview regarding eating behaviors; and (3) a questionnaire outlining current smartphone and app use and paper prototyping activity.

#### Phase 2: Preliminary End-User Testing

An early pilot app was developed using information from both the initial scoping review [2] and preliminary analyses of the data collected in phase 1 (please see images supplied in Multimedia Appendix 1). Features and content of the early pilot app relate to the constructs of the Theory of Planned Behavior (TPB): attitudes, subjective norms, and perceived behavioral control [19,20]. The app contained a log-in screen for the user to enter individual information that could be used to estimate energy balance and associated weight changes. Screens for selecting and tracking regularly consumed drinks and progress screens presenting information about the sugar and energy consumed in the past month from drinks were included in an effort to impact control beliefs, and therefore, positively influence users’ perceived behavioral control over SSB consumption [19]. Team challenges aimed at supporting nutrition improvement were incorporated to influence normative beliefs, and therefore, adjust subjective norms around the consumption of SSBs [19]. Finally, a quiz-style game was included with the aim of positively influencing attitudes toward SSB consumption, and therefore, behavioral beliefs and intention to change [19,20]. The preliminary end-user testing phase aimed to trial the functionality and acceptability of the developed early pilot app. It included the following 2 components: (1) think aloud prototype test and (2) user satisfaction semistructured interview.

### Setting

The study was conducted in 2 RICs in the Northern Territory, Australia. The communities were purposively selected to represent diversity in culture, geographic location (coastal vs inland), and remoteness (339 and 890 km from the capital of the Northern Territory, Darwin). Community authority or cultural safety groups within communities were approached and provided with information on the project to ensure community support and advice on the study process. Following approval from the relevant community authority or cultural safety group, project information was provided to key community leaders, services, and groups. Data collection for the formative research phase took place between September and November 2016, with end-user testing in December 2016.

### Recruitment and Sampling

Purposive sampling was used to recruit 10 participants from each community for each research phase, with researchers positioning themselves and study materials at prominent locations in the community, including the community store and recreational areas. Participants who completed phase 1 were invited to also complete phase 2; however, new participants were recruited for phase 2 when existing participants were unable to be followed up. Eligible participants were Aboriginal and/or Torres Strait Islander adults aged between 18 and 35 years who were active smartphone users (ie, use a messaging/chat system at least 3 times per week and/or other phone features) and who had planned to reside in the community until December 2016. Sampling aimed to recruit a balance of genders and participants of a variety of ages, languages, and family groups.

### Data Collection and Analysis Methods

#### Formative Research

The formative research phase involved the following 3 components: a think aloud shop, a semistructured interview exploring food behaviors, and a brief questionnaire examining smartphone use with a paper prototyping activity [21]. Two researchers collected data (LJ and BC), 1 researcher from each gender to work with participants of the same gender to ensure cultural norms were respected. The 3 parts of the formative research were carried out sequentially with each participant in 1 session, and sessions ranged from 20 to 60 min in length. A local interpreter was available to assist when required. Participants chose the location for each session and sessions were completed individually, in pairs, or in groups at the participants’ discretion. Additionally, during the interviews, many participants were accompanied by children, partners, or friends. All sessions were audio-recorded, transcribed verbatim by 1 researcher (LJ), de-identified, and imported into NVivo 11 (QSR International, Doncaster) for analysis. Analysis varied by study component.

##### Think Aloud Shop

The think aloud shop activity was included to collect data related to the determinants of food choice for young adults living in RICs. These data were collected to identify key nutrition features and content for the app. Think aloud methods are increasingly used in nutrition research to gain insight into food decision-making processes [22-24]. These methods are based on the foundational work of Ericsson and Simon [25] and involve participants completing a task while verbally narrating their thoughts to elicit the sequence and information involved in processing the task. In nutrition research, the original method from Ericsson and Simon is typically adapted to suit the research aims and setting, the setting often being an accompanied shop [26]. While an accompanied shop scenario was considered ideal by the research team, this was logistically unachievable in the RIC setting for a number of reasons that are elaborated in the Discussion. As such, participants were asked to shop using a paper supermarket catalogue while thinking aloud. The catalogue used for the think aloud task extensively advertised SSB in prime positioning, as well as other healthier alternatives such as bottled water. A modified version of the think aloud training described by Barnett et al [22] was used to familiarize participants with the method [27,28]. Standardized prompts such as “what are you thinking?” were used to remind participants to think aloud when they had fallen silent [22]. The think aloud task was completed when participants stated they were finished. Thematic analysis of the think aloud shop transcripts focused on identifying reasons for food choice. Two researchers (LJ and BC) separately coded the transcripts and developed a list of themes related to food choice emerging from the data. The separate lists were then merged and definitions for each theme were agreed upon and defined in a codebook. Using this codebook, 2 researchers (LJ and ET) then recoded all transcripts, ensuring analyst triangulation [29]. A high level of agreement in coding was achieved, and disagreements were discussed until a consensus decision was reached.

##### Semistructured Interview

The semistructured interview (Multimedia Appendix 2) aimed to collect data related to eating behavior and food attitudes. These data were collected to determine approaches to app design to best support behavior change in this population group. The interview schedule was designed using the constructs of the TPB [19,20]. The questions explored social identity associated with food, self-efficacy, knowledge, and intention to make dietary changes, and supports and barriers to previous attempts to changing eating behavior. Analysis of the interview transcripts was carried out by 2 researchers (LJ and ET). The transcripts were segmented into sections based on question, and the responses to each question summarized. Most participants provided yes/no responses and also elaborated with further explanation. Summaries of the responses for each question were synthesized and grouped according to the constructs of the TPB for presentation.

##### Smartphone Use Questionnaire and Paper Prototyping Activity

**Figure 1:**
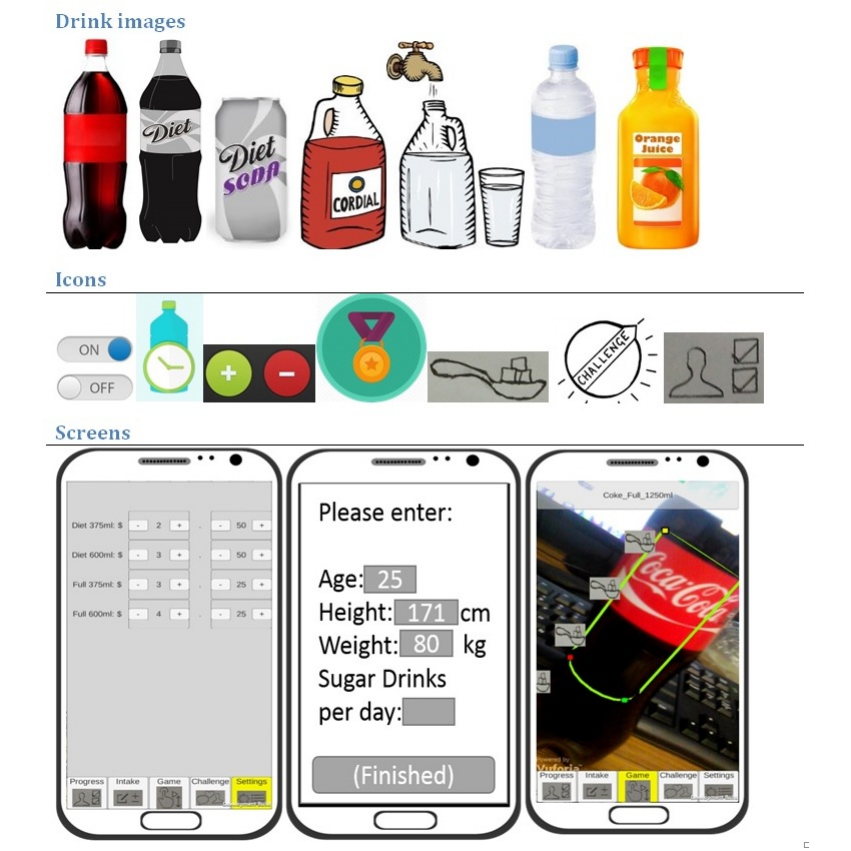
Example trigger materials for paper prototyping activity.

A questionnaire examining current smartphone and app use elicited key data to inform foundational app functionality (Multimedia Appendix 3). Questions were developed by research team members with experience working in RICs to specifically target known phone behaviors unique to the RIC setting. The questionnaire was administered verbally to ensure adequate understanding of all questions. The paper prototyping method has been used in previous app development scenarios [30] to involve target users in the development of app features and styling. This method involves the researcher using trigger materials (eg, images showing features and capabilities of health-related apps) to encourage participants to build an app prototype. In some cases, due to constraints related to location, environmental factors, and the presence of young children, it was necessary to modify the paper prototyping activity. In these instances, the activity was altered to instead present participants with different trigger materials (see examples in Figure 1) and ask for their preferences with reasoning. As the paper prototyping activity was conducted similarly to a structured interview, transcripts were analyzed by question, and responses to both the paper prototyping activity and questionnaire were aggregated and summarized numerically by 1 researcher (LJ). Qualitative data from the paper prototyping activity has been added where appropriate.

#### End-User Testing

End-user testing involved the following 2 components: usability testing of the early pilot app using the think aloud method [25], followed by a brief interview regarding comprehension and satisfaction.

##### Think Aloud Usability Testing

Think aloud is a common approach to assess software usability [31,32] and app prototype testing [21]. Think aloud sessions were conducted with 10 participants from each community as Nielsen [31] showed that there are diminishing returns for each think aloud test conducted beyond 7 participants in the context of software usability testing, and this is consistent with other studies [33,34]. Participants were familiarized with the think aloud method using the same procedure as in the phase 1 think aloud shop. Participants were then asked to download the app and carry out a series of tasks using the app while thinking aloud. This involved each participant working through each screen of the app (eg, “log-in screen” and “games screen”) and being prompted to think aloud while doing so. Sessions were audio-recorded and researchers noted actions and issues with usability, comprehension, and satisfaction. The protocol identified that, if necessary, participants would be asked to elaborate on these issues in a short interview after all tasks were complete, not while thinking aloud, to preserve an uninterrupted stream of information processing [25].

##### Interview

User satisfaction was then assessed using a structured, audio-recorded interview with the end-user participants (Multimedia Appendix 4). This interview involved verbal administration of a modified version of the System Usability Scale (SUS) [35] as it has been shown to be a robust tool across many user interfaces [36] and used in other app development projects [30,37]. In addition to SUS, questions were adapted from the Computer System Usability Questionnaire [38]. Modifications to these standard questionnaires were necessary to ensure comprehension for participants in the RIC setting. Modifications included exchanging technical words, and those that experts suggested would not be comprehended, with words that would be understood (eg, “this system” was replaced with “the app” and “well-integrated” was replaced with “worked well together”). The researcher assessed comprehension when administering the instrument and further rephrased questions to ensure comprehension as necessary.

Analysis of the think aloud usability testing and user satisfaction interview transcripts focused on the themes of usability, comprehension, and satisfaction, and was carried out by 1 researcher (ET). In addition to the transcripts, the researchers’ notes on these themes from testing sessions were also included in the analysis. Data were organized according to app screen and synthesized under the main themes for presentation here.

## Results

### Participant Details

Ten participants from each of the 2 communities completed the formative research phase; 4 also completed the end-user testing phase, as did 16 new participants. Participant characteristics are provided in Table 1.

The following sections report the findings for each of the 2 research phases, organized by study component.

### Formative Research

The formative research phase collected data related to drivers of food choice, eating behavior, attitudes toward dietary change, and smartphone use to inform app content and functionality.

#### Think Aloud Shop

Eight major determinants of food choice were identified through the think aloud shop activity. Taste was the most frequently vocalized reason for food choice, with 17 participants (85%) referring to positive taste attributes (sweet, salty, crunchy) on 50 occasions throughout the sessions. Family was another key driver, with children’s preferences frequently motivating choice. Health also appeared to be front of mind for 15 participants (75%); however, sometimes health was considered but not acted on; for example, “It’s got nice flavor, I know it’s got too much salt and fat, I know that, I just get ’em when we are hungry.” For younger participants (18-25 years), the ability to share or use food during social activity was an important consideration and often motivated choice. Nine (45%) participants made food choices driven by price during the shop, with foods either considered to be cheap or of good value.

**Table 1:** Participant characteristics.

Characteristics		Formative research (n=20) n (%)	End-user testing (n=20) n (%)
**Gender**			
	Female	10 (50)	11 (55)
**Age in years**			
	18-20	6 (30)	0 (0)
	21-25	5 (25)	5 (25)
	26-30	5 (25)	7 (35)
	30-35	4 (20)	8 (40)
**Family situation**			
	Children present	7 (35)	5 (25)
	Partner present	1 (5)	4 (20)
	Partner and children present	1 (5)	5 (25)
	Friends/family present	10 (50)	1 (5)
	Alone	1 (5)	5 (25)

Convenience was another factor considered by 6 participants (30%), and this related to either the ease and speed of meal preparation, or alternatively that the food was useful for taking on bush trips (eg, conveniently packaged and heat tolerant). The final major factors motivating food choices were familiarity with a product and repeatedly purchasing it as part of a regular shop and the longevity of a product in the sense that it could last until the next shop or pay.

#### Semistructured Interview

##### Control Beliefs and Perceived Behavioral Control

When discussing making changes to their eating habits, 10 of 19 participants (53%) would like to involve family and friends. Furthermore, 9 of 19 participants (47%) preferred to make changes alone; however, with 1 participant demonstrating her reasoning using the example of smoking:

Cause some families are smokers too and instead of encouraging you to stop smoking, they’ll keep on smoking beside you and making you want to smoke, cause that’s what happening to me now.

Most participants (16/20, 80%) identified that there are drinks that they would find difficult to give up, soft drinks (defined here as carbonated drinks, either unsweetened, sweetened, or artificially sweetened) being the main type of drink mentioned (14/20, 70%). However, others considered changing their drinking habits to be easy, particularly when they had a strong motivation:

Participant: I quit [cola soft drink brand] 2 weeks ago...Cause I just had too...I’m always thinking about the other two [her children].

Researcher: What did you change to?

Participant: [diet cola soft drink]

Researcher: Was that an easy change or hard change?

Participant: Easy.

One participant mentioned that flyers and reminders around the community would help her stay on track with dietary changes.

##### Behavioral Beliefs and Attitudes

Many (11/20, 55%) participants did not believe that what they eat affects their body and mind, and these same participants also did not worry about their dietary habits. Seven of these same participants also did not find anything about food and drink interesting or worth learning more about:

I know about the drinks because I went to the factory. I went when I was, we went with school trip. I know about drinks how many sugar they got and how many thing they got [sic]. I know about that.

The other 9 of the 20 participants (45%) did, however, believe that dietary habits impacted health, reporting worrying about food choices:

Sometimes I worry to eat good foods...we might get sick like from the other foods.

This group did find information about food and drink interesting and wanted more specific information related to recipes, cooking, and food composition:

I would, yeah, be happy to learn more especially when comes down to recipes and mixing and yeah that sort of stuff. Pretty much basic but there is more to it.

Other responses indicated a need for more resources related to food choice:

Yeah, to you know when you go out maybe or when you’re at the shop or somewhere and you need something that you need, and you know with the sheet and show you what you can eat.

##### Normative Beliefs and Subjective Norms

When discussing foods that made participants feel strong and proud, most described bush foods (“bush tucker”), fishing, and hunting for animals such as barramundi, turtle, bush turkey, and kangaroo with family and friends. In contrast to these foods, some participants discussed drinking sugary drinks:

I’m normally addicted to um [cola soft drink brand]...I drink [cola soft drink brand] whenever I’m out.

Water and fruit was also frequently mentioned. In terms of learning about healthy eating, most participants (7/12, 58%) reported being taught the most from their family, whereas others specifically mentioned the health service (2/12, 17%), their friends (1/12, 8%), the store (1/12, 8%), and their work (1/12, 8%).

##### Behavioral Intention

Participants were evenly split regarding whether they would like to change their eating behaviors in any way (yes, n=7; no, n=7). However, it was clear that “change” was a difficult concept for these participants. Translators highlighted that it was hard to explain to participants, and 1 participant verbalized the difficulty when answering the question:

That’s a hard one. But if I were to change someone, hang on, how do I put it? Sorry what’s the question again?

Despite this, most participants described having attempted dietary change before (12/20, 60%), typically motivated by health, as shown in the quote below:

I want to go back ’cause I’m overweight. That’s what I want to do...Make myself lighter, cause when I was light I was alright, I can do everything, you know, do anything. But at the moment I’m stuck with my fatness and I need to lose it all

#### Smartphone Use Questionnaire and Paper Prototyping Activity

The details of smartphone and app use can be found in Table 2. Generally, the brand of phone was determined by factors outside the participant’s control, such as it being the only brand available at the store at that time, or it was a gift. Participants most commonly used their smartphones for SMS text messaging (short message service, SMS) or calls (19/20, 95%) and games (16/20, 80%), with less common uses being to watch movies (8/20, 40%), do banking (5/20, 25%), access Centrelink (5/20, 25%), play music (4/20, 20%), and access email or social media (3/20, 15%). More than half of the participants (11/20, 55%) did not loan their phone to others, whereas 7/20 (35%) did loan their phone to family, typically their children, and 2 of 20 (10%) allowed others to use their phone for calls. The majority of participants used multiple languages when texting (Table 2), such as Rembarranga, Kriol, Warlpiri, and Guringji. Only a few participants owned other information technology (IT) devices: 3 of 20 (15%) owned iPads and 2 of 20 (10%) owned laptops. Some participants referred to owning tablets or laptops that were no longer functional. Games, YouTube, and banking apps were the most common apps used, and additional details of app use can be found in Table 2.

During the paper prototyping activity, participants suggested ideas for a health app for their community, with the most common ideas being an app on how to be strong, healthy, and fit (4/20, 20%), an app to provide information on good/bad foods (4/20, 20%), a food/health games app (3/20, 15%), a quiz app (2/20, 10%), and an app with cooking ideas/recipes (2/20, 10%). A number of participants elaborated as follows:

A health app, maybe in [local language] too, with a quiz game you know in [local language]. Talk about...just a little guessing game how much you reckon in [cola soft drink brand] an all that, ’cause asking them is trying to get an idea of that person in answering back.

So, if it’s going to be health wise, it’s just something to do with fitness, like marathon running or footy game or something, something to do with sports with fitness.

Specific app features that participants wanted within an app included games (3/11, 27%), audio/music (3/11, 27%), videos (2/11, 18%), and e-stories (2/11, 18%). One participant preferred Aboriginal art and the colors of the Aboriginal flag (red, yellow, and black) to be featured.

The following sections describe findings relevant to specific app screens. As this activity was participant-led as much as possible, not all participants contributed to all sections.

##### Log-In and Profile Screen

Only 1/20 participant indicated they were happy to sign in via Facebook, with most other participants not holding a Facebook account. Although 9 participants had no objection to include their height and weight in their app profile, many did not know their anthropometric measurements and therefore suggested these figures would have been guessed. Three participants wanted to keep their height and weight information private. Four participants liked the idea of inspirational images and suggested these could include sport, food, or personal photos.

##### Drink Selection and Recording Drinks Screen

Six participants said they would like to use an app to record their drinks. A number of participants elaborated on this question: 1 participant would like to record their drinks for a week, another 1 day at a time, 1 participant suggested this would be easy, and another mentioned it would be difficult to remember to do but would be useful if you remembered. Two participants would be happy to share their drink history; however, 1 participant was asked if they would still want to share even if they had consumed “bad” drinks and they responded, “no...some...I will share with my closest friends.” Most participants (4, 57%) preferred drinks to be displayed as a picture of a bottle with words. Participants preferred grouped icons to a scroll-through function for selecting drinks.

**Table 2:** Characteristics of smartphone and app use.

Characteristics					Participants (n=20) n (%)
**Characteristics of smartphone use**					
	**Brand of smartphone**				
		Telstra (Android OS^a^)			8 (40)
		Samsung (Android OS)			5 (25)
		iPhone (iOS)			3 (15)
		Huawei (Android OS)			4 (20)
	**Length of current phone ownership**				
		<1 month			3 (15)
		2-5 months			5 (25)
		6-12 months			1 (5)
		1-2 years			3 (15)
		2-3 years			6 (30)^b^
		“long time”			2 (10)
	**Frequency of carrying phone**				
		Always			14 (70)
		Sometimes			6 (30)
	**Daily usage of smartphone**				
		<once per day			2 (10)
		>once per day			17 (85)
		No response			1 (5)
	**Languages used when texting**				
		Mix of local language and English			9 (45)
		Mostly English, some local language			6 (30)
		Only English			3 (15)
		Pidgin English			2 (10)
	**Internet use^c^**				
		Mobile data			7 (35)
		Wi-Fi			5 (25)
		Both			4 (20)
		No Internet use			4 (20)
**Characteristics of app use**					
	**Facebook account ownership**				
		Yes			9 (45)
			**Facebook app use**			
				Yes	1 (11)
				No	4 (44)
				Undetermined	4 (44)
		No			11 (55)
	**Previous use of social media or apps to assist in dietary change**				
		Yes			0 (0)
		No			20 (100)
	**Apps used by participants^d^**				
		Games			16 (80)
		YouTube			7 (35)
		Banking			5 (25)
		AFL^e^			4 (20)
		Music			4 (20)
		Centrelink^f^			3 (15)
		Maps			3 (15)
		Email			3 (15)
		Snapchat			2 (10)
		Gumtree^g^			2 (10)

^a^OS: operating system.

^b^Two participants had recently lost their phones and were planning to purchase a new one, but had owned the phone for 2 to 3 years before losing it.

^c^One interpreter corrected participants when they mentioned they used Wi-Fi; it seemed that participants were unclear about the difference between mobile data and Wi-Fi. Only 1 participant clearly described using both.

^d^The researchers observed that with some participants it was unclear if the apps mentioned were indeed apps or accessed via an Internet browser; for example, YouTube and email.

^e^AFL: Australian Football League.

^f^Centrelink is a department of the Australian Government Department of Human Services that administrates welfare and social support payments.

^g^Gumtree is an Web-based platform for selling used goods.

Accurately recording drinks consumed was highlighted as a problem as the researcher identified that 3 participants were sharing bottled drinks with family members during the activity. This was discussed with 1 participant as shown in the following quotes:

Participant: Well if it was a little can I would maybe give them about that bit...Just a few sips and if it was bottle sometime I would only have half or just store it up for myself.

Researcher: Would that make it hard if you were recoding your drinks, do you think that would make it hard sharing and just drinking half?

Participant: I reckon yeah. Yep

Researcher: Do you think, how can we get around that in the app?

Participant: Oh that’s a hard one now, maybe if there was a sip included, or how many sip you reckon; one, two sips. Or if it’s like scull [ie, drinking a lot very quickly], then scull.

##### Feedback/Progress Screen

Participants suggested to have their progress displayed in body weight gain (kilograms) prevented, money saved, and volume of sugar intake prevented. Most participants (10/16, 63%) preferred sugar to be displayed as teaspoons, rather than bags (3/16, 19%), cups (1/16, 6%), sugar cubes (1/16, 6%), or as a percentage of volume/energy (3/16, 19%). Three participants thought a leader board was a good idea, with 1 noting this could be based on water consumption.

##### Challenges Screen

Both participants who were asked said they would like to complete a challenge and notify their friends and family. They both preferred to select from a list of challenges rather than develop a challenge themselves, participate “sometimes...yeah when I feel like it,” and suggested prizes could be real money (AU $50-100). One participant suggested some motivational messages that would be provided by the app, which were as follows:

To keep going like with what we’re doing, like to change.

Say to me to be strong and keep going.

Give my kids change a bit [suggest to me to implement changes with my children], like for me as well.

From the 3 components of the formative research phase combined, a list of commonly selected beverage choices was compiled and is presented in Table 3.

**Table 3:** Common beverage choices in the remote Indigenous communities studied, ordered by frequency of mention.

Beverages		Formative research component in which the beverage was identified		
		Think aloud shop	Eating behaviors interview	Paper prototyping activity
**Soft drinks**				
	Cola (Coke, Pepsi)	Identified	Identified	Identified
	Lemonade (Sprite)	Identified	Identified	Identified
	Lemon soft drink (Lift, Solo)	Identified		
	Diet cola (Diet Coke, Coke Zero, Pepsi max)	Identified	Identified	Identified
	Orange soft drink (Fanta, Sunkist)	Identified		
	Passionfruit soft drink (Pasito, Passiona)	Identified	Identified	
	Raspberry soft drink	Identified		
	Citrus soft drink (Mountain Dew, 7-Up)			Identified
	Diet lemonade	Identified		
	Flavored mineral water (Deep Spring)			Identified
	Diet creaming soda soft drink	Identified		
	Ginger beer			Identified
	Diet lemon soft drink	Identified		
	Soda water	Identified		
**Other drinks**				
	Water	Identified	Identified	
	Tea	Identified	Identified	
	Coffee	Identified	Identified	Identified
	Fruit drink	Identified	Identified	Identified
	Orange juice	Identified	Identified	Identified
	Cordial	Identified		
	Sports drinks (Powerade)		Identified	
	Apple juice	Identified		Identified
	Milk/Up and Go		Identified	Identified

### End-User Testing

**Figure 2:**
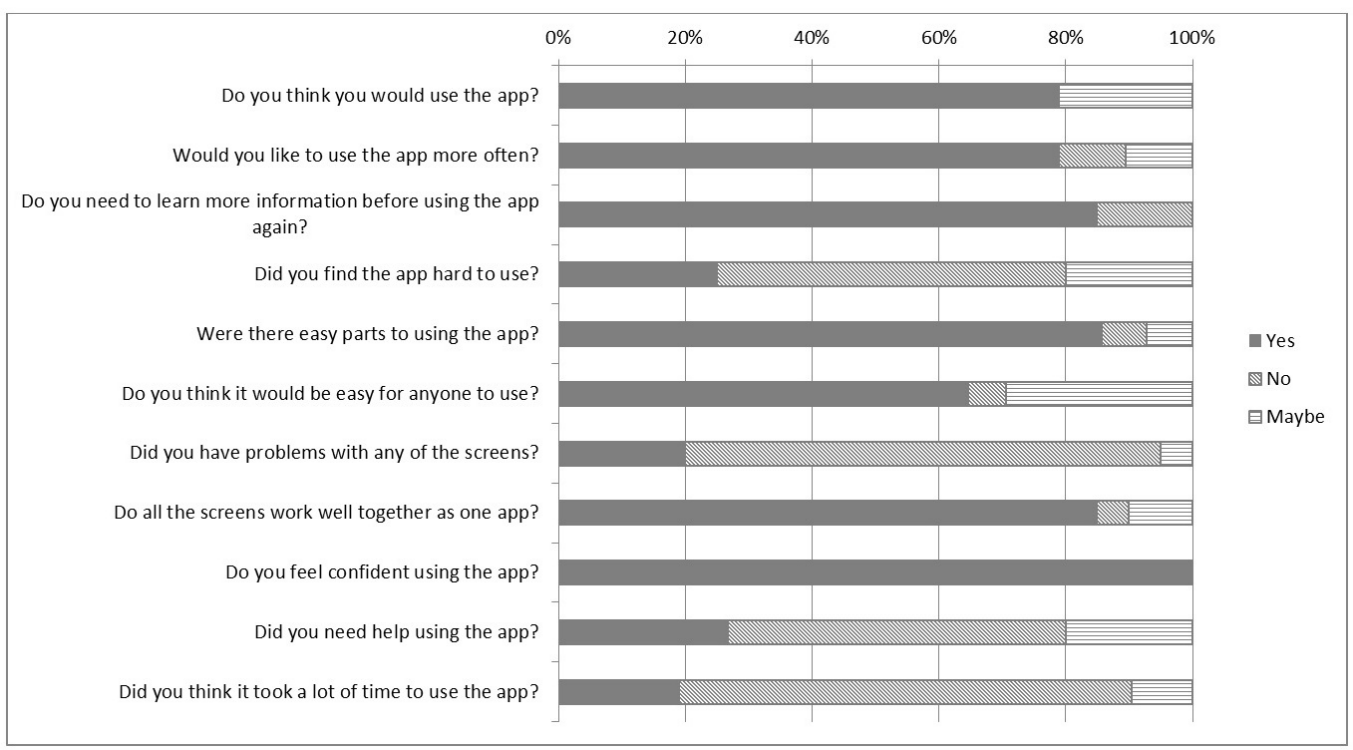
Responses to end-user testing interview questions.

The usability and participant comprehension and satisfaction with the early pilot app were assessed in the end-user testing phase. Briefly, screens included general log-in and profile screens, screens for recording SSB intake (drinks selection and recording screens), screens demonstrating progress/feedback related to SSB consumption, a screen enabling users to complete challenges related to reducing SSB consumption, and games (Multimedia Appendix 1). Here, we report findings from the think aloud testing most translatable to other development projects, and the responses to the user satisfaction interview are graphically presented in Figure 2.

#### Think Aloud Usability Testing

Overall the app was considered usable, albeit slow, given that it was an early prototype. Besides the issues arising from participant unfamiliarity with apps in general (eg, difficulty using scroll functions and navigating using a ribbon menu), many participants struggled to grasp the concept of the app. However, once it was explained it was well received and many participants commented that it would be of great benefit to their community, especially for people with type 2 diabetes and children. Generally, there was an app component that every participant enjoyed, whether it was the quiz, challenges, or simply entering information about consumed drinks. Most participants reported their satisfaction would be increased with additional use of sound within the app, including talking voices providing information, explanations, and stories; music; and notification sounds such as “bing.” One participant noted that more role modeling through the use of images with people choosing different drinks would be motivational. A number of male participants suggested that enhancing links to sport and bush tucker would increase interest. Additionally, many participants reported it would be more useful if the app scope was extended to include food. Finally, many participants referred to wanting to include family in some way within the app, especially their children.

The following sections describe findings relevant to specific app components that may be transferrable to other app development projects.

##### Log-In and Profile Screen

It was confirmed that using a Facebook log-in was not feasible as again there were a low number of Facebook users in the sample. In addition, self-reported anthropometric information (for body weight change and energy balance feature estimates) was unlikely to be accurate. Most participants found profile setup difficult due to the use of scroll functions. Some participants reported that having to go in and out of settings to change profile information, including regular drinks, made the app confusing and frustrating to use. Notifications were also not well understood as a concept, but once explained participants commonly set their ideal notification receipt time as midmorning and were pleased with the customizability of this feature.

##### Drink Selection and Recording Drinks Screen

Again, many participants struggled with the concept of selecting “regular” drinks and then adding daily consumption in a later screen (see Multimedia Appendix 1, screens 3, 4, and 5). They appeared to think they had to have consumed a particular drink that day to add it, rather than simply selecting usual drinks consumed over an extended period. Many suggested the addition of hot drinks, such as tea with sugar, was needed to make app use worthwhile. Generally, most participants found it easy to differentiate (intentionally nonbranded) drink types based on shape (can, small, or big bottle) and color (see Multimedia Appendix 1, screen 3), but many suggested including logos would further assist recognition.

##### Feedback/Progress Screen

Progress reported as teaspoons of sugar was well received; however energy (presented as “kJs”) was not understood as a concept, and some participants wanted to see total drinks consumed in bottles/liters. Presenting progress in monthly increments did not seem to be useful, with shorter time periods preferred, such as daily or weekly. It was again confirmed that many participants would have liked to see progress reported as change in body weight, although it was unclear whether they wanted this projected based on energy within drinks, or if they wanted to track their actual body weight. Some suggested a talking voice to explain their progress would assist comprehension. Finally, some participants wanted suggestions of better choices at the shop based on their entries.

##### Challenges Screen

The challenges were initially not well understood as a concept, but once explained participants were eager to try them. They thought presenting images with the challenges was fun and enhanced comprehension. As with the progress screen, many wanted more explanation as to why these challenges would help, preferably verbally. Participants were seeking a greater response from the app upon reporting completion of a challenge; again noises, flashing, ticks, and visual messages such as “good work.”

##### Games

Overall, the quiz feature was well comprehended, worked well and had high reported satisfaction. Some participants wanted feedback on their guesses to be clearer, including noises as well as visuals such as a tick or cross. A minority did not interpret the subtle color change of the correct answer as feedback (Multimedia Appendix 1, images 7 and 8). Many participants reported that including more games such as the quiz would increase their interest and frequency of use of the app.

## Discussion

### Principal Findings

This study used a consultative user-centered approach to identify key considerations for app design that can be used to inform app development for disadvantaged and nonurban populations, specifically young adults living in RICs. A number of important insights regarding app functionality have been illuminated, as have considerations for features and content to ensure user engagement and comprehension in disadvantaged, nonurban populations. Additionally, important learnings related to carrying out best practice app development research have been highlighted through the process of conducting this research. These findings are discussed in the context of prior work in the following sections, and Textbox 1 outlines concrete recommendations arising from the research that are translatable to a wide range of app development projects.

### Comparison With Prior Work

The findings support previous work emphasizing that a user-centered design process is critical to developing a functional app [39-41]. This is starkly demonstrated here, where despite high rates of Facebook update in the Australian population, only 1 participant reported the willingness to use a Facebook log-in to access the app in the formative testing, and no participants had a functioning Facebook account to log-in with during the prototype testing. Use of Facebook log-ins is routine in app development, thought to minimize user burden. However in this setting exclusive use of a Facebook log-in would have excluded most of the target user group. Similarly, it was found that the types of phones used by participants were determined by those available in the RIC store; thus, initial groundwork to establish what these are is essential for optimizing apps for these target users. Additionally, the choice to develop a mobile app was reinforced as few participants had either access to other IT devices or consistent Internet availability for Web browser–based interventions that require constant Internet connection, unlike a mobile app. Therefore, despite the increased use of smartphone technology in RICs [5], it was established that characteristics of smartphone use are currently different in a number of small but critical ways when compared with urban, young adult populations. These findings, therefore, highlight that thorough formative research is vital for app development projects in other disadvantaged, nonurban populations.

Strategies for fostering engagement with both apps and dietary change in a disadvantaged, nonurban population were also identified. The participants in this study were evenly divided in their desire to involve family and friends in any potential dietary change.

Previous literature has shown that competition, team, and social interaction were useful for motivating app engagement across a variety of population groups [30,37,42-46]; however, research with young adult populations emphasizes this must be voluntary [46-49]. Therefore, providing users with the *option* to include others through an opt-in approach to sharing and leader boards, and making both personal and group challenges available, is critical to ensure those users who do not find social support within the app as necessary for change are not alienated from the app. It also appeared from the formative semistructured interview responses that a large number of participants were not aware of the need to, or not motivated to, change their SSB consumption behaviors. While the behavioral theory used in designing the study, and therefore, the pilot app was TPB, these data emerging from the formative work are usefully explored using the Transtheoretical Model of health behavior change [50]. It appears that half the participants were in a “precontemplative” stage of change, whereas the remaining participants were at other stages along the behavior change pathway, potentially making them more open to, and therefore engaged with, app content.

**Recommendations for app development projects targeting disadvantaged and nonurban populations arising from this study.**

Project development recommendations

- Prioritize the conduct of comprehensive formative research in project planning
- Work in development teams that include members with in-depth local knowledge
- Utilize mixed data collection methods (quantitative and qualitative) and triangulate methods where possible
- Consider the potential for the availability of technology and patterns of use to differ greatly in the target group compared with urban populations, and therefore, include this in formative data collection

App development recommendations

- Include nonwritten communication strategies extensively throughout the app to aid comprehension (eg, audio of talking, inclusion of audio cues such as “beeps” and “dings”)
- Include colorful, animated feedback throughout the app to foster engagement
- Include local jargon where possible (eg, in this case, “sip” and “skull”)
- Include games and tailor these to the target population through language, color, sound, and content
- Include only tangible health language (eg, spoons of sugar, not calories)
- Ensure social aspects of the app are opt-in
- Ensure the app is disseminated in a supportive context (eg, through a health provider)

This suggests that it would be useful to use the Transtheoretical Model alongside TPB to structure app content and features to cater for users at all stages of the behavior change cycle, particularly features that draw participants in, despite a lack of intention to change target behaviors. One option to engage this group of users is the inclusion of games with an inherent educational element, with games commonly being used in apps targeted toward low socioeconomic status and adolescent populations to enhance engagement [2,51,52]. During all stages of this research, it was demonstrated that even simple quiz games were enjoyed, consistent with findings from others [53-55], and in this, participant group games were the second most common reason for phone use. Thus, the inclusion of games in any app targeted within RICs or similar disadvantaged, nonurban populations appears to be essential, especially for initiating and maintaining engagement of users in a precontemplative stage of change. Other app content such as feedback and progress information will be more useful to users in “determination,” “action,” and “maintenance” stages of change.

A key advantage of a user-centered design approach is to tailor apps to specific target populations to enhance not only engagement but also comprehension [2,40]. The findings suggest audio features, sounds, and voice-delivered explanations would augment comprehension across all app screens in this user group. It was also evident in the prototype testing that participants struggled to grasp the overall concept of the app before it was explicitly described. This may be explained by the fact that the participants were presented the pilot app somewhat out of context; for example, it was not suggested by a friend or health worker as might be the case in a real-world scenario. This suggests that dissemination of future apps needs to be contextually embedded, with many potential avenues available. Apps could be presented and explained to users at clinic visits [56], through sporting team coaches or schools [57], or as part of larger interventions [58]. Another design consideration related to comprehension was the need to streamline the app as much as possible. This is consistent with previous literature suggesting that complex setup and log-ins inhibit use [46,48,49,59]. As the need for streamlining potentially presents a competing priority to the aforementioned need for customizability, piloting of apps with target users will be essential to achieve a successful balance.

A number of unique challenges impact everyday life in RICs and provide context for these findings. Many RICs are serviced by a single food store that sells a limited range of packaged, fresh, and takeaway foods [60]. These stores often have restricted opening hours, closing at late notice for important community events. Similarly, idiosyncratic behaviors related to smartphone use result from the nearest location to buy and service IT devices often being hundreds of kilometers away. The social dynamics of RICs too are not comparable with the urban environments in which many apps are developed and tested, as many communities are composed of only a few hundred people [61], with many related. Finally, many Indigenous Australians enter parenthood earlier than nonIndigenous Australians [61,62], dramatically altering the outlook, priorities, and eating behaviors of this young adult group compared with their nonIndigenous counterparts. As such, the value of this research extends beyond this project alone to others who are exploring eating behaviors and smartphone use in disadvantaged, nonurban settings, and the user-centered development approach employed in this work is a major strength of the research.

### Limitations

These unique qualities of RICs, however, impacted how best-practice app development methods could be implemented, generating limits to the generalizability and comparability of the research findings but strengths regarding validity and authenticity of the data. Given that for most participants English was a third, fourth, or fifth language, with much of the terminology used in the context of app development simply untranslatable, the modification of the end-user testing methods enabled participant comprehension, and therefore, the collection of useful and insightful data; however, responses cannot be scaled and compared with similar projects. Similarly, the limited opening hours of the 1 community store, combined with the small population resulting in participant discomfort with thinking aloud in the store, meant modification of the think aloud shop activity was required. While this prevents nuanced environmental impacts of the store environment from being included in the drivers of food choice, it ensured the data collection was completed at the participant’s convenience, comfort, and in relative privacy, thus enhancing data quality overall. In addition, a major strength of the project was the triangulation of methods and combination of qualitative and quantitative methods and analysis [29]. For example, without all 3 of the components in the formative research phase, the comprehensive list of SSBs consumed in RICs (Table 3) could not have been generated. This provides an important lesson for future research. Therefore, the detailed methods and research tools provided in this report may serve as a roadmap for future user-centered app development projects.

### Conclusions

This research provides critical insights for the development of apps targeted toward young adults in disadvantaged populations and nonurban settings. It also serves as a framework for future app development projects using a consultative user-centered design approach and supports calls for the increased use of this strategy in projects developing apps for health behavior change.

## Appendix

Multimedia Appendix 1 Screenshots of the app.

Multimedia Appendix 2 Interview guide—eating behavior and food attitudes.

Multimedia Appendix 3 Smartphone and app-use questionnaire.

Multimedia Appendix 4 Structured phone interview schedule for stage 2 app testing.

## Abbreviations

IT: information technology
RIC: remote Indigenous community
SSB: sugar-sweetened beverage
SUS: System Usability Scale
TPB: theory of planned behavior
