# Supplementary material for: A Smartphone App to Reduce Sugar-Sweetened Beverage Consumption Among Young Adults in Australian Remote Indigenous Communities: Design, Formative Evaluation and User-Testing
Source: JMIR Mhealth Uhealth. 2017 Dec 12;5(12):e192. doi: 10.2196/mhealth.8651 (PMC5743922; doi:10.2196/mhealth.8651)

Multimedia Appendix 1

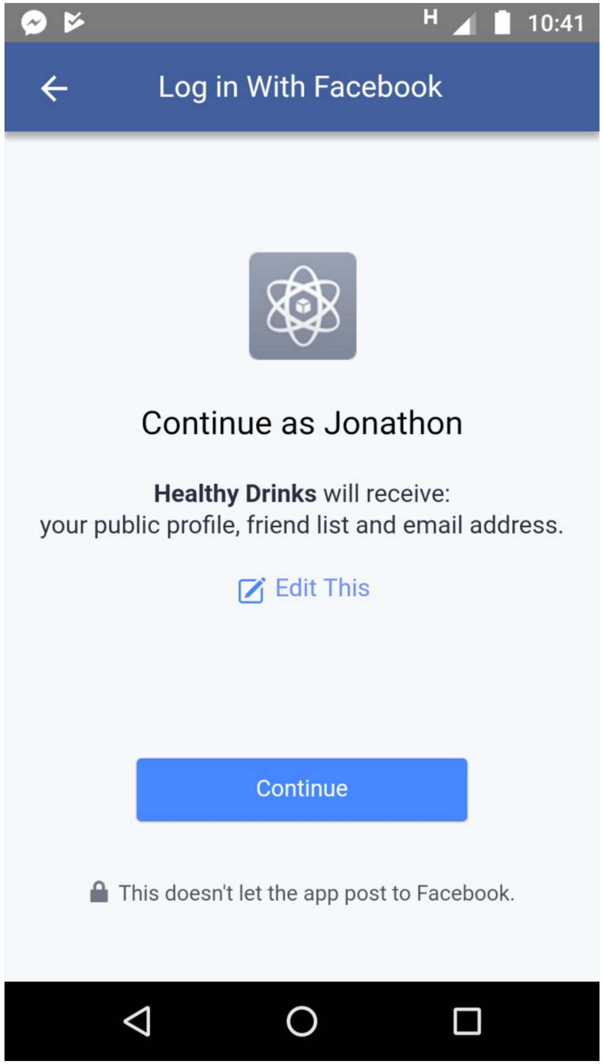

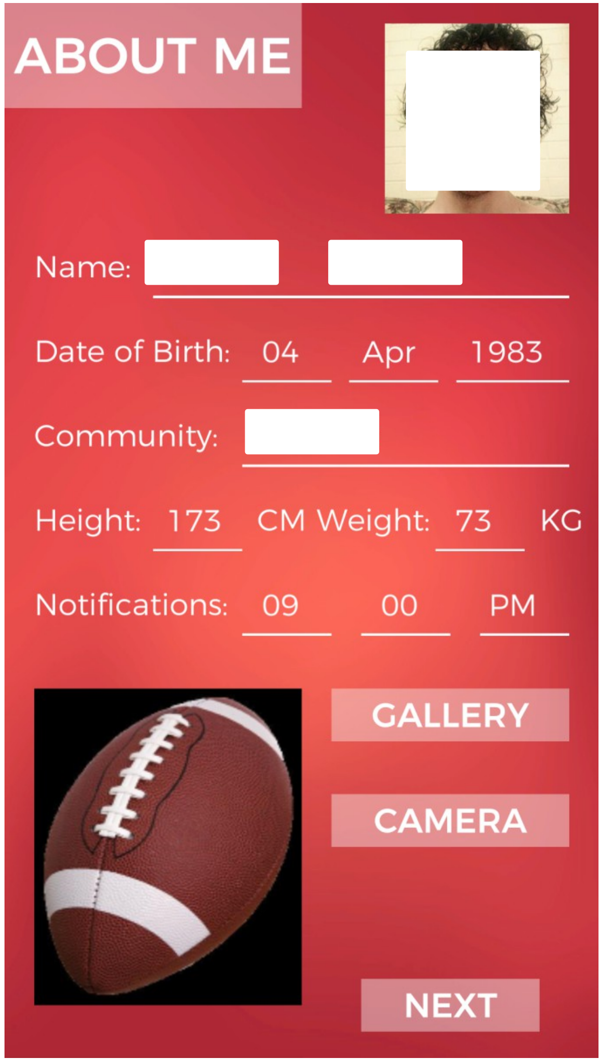

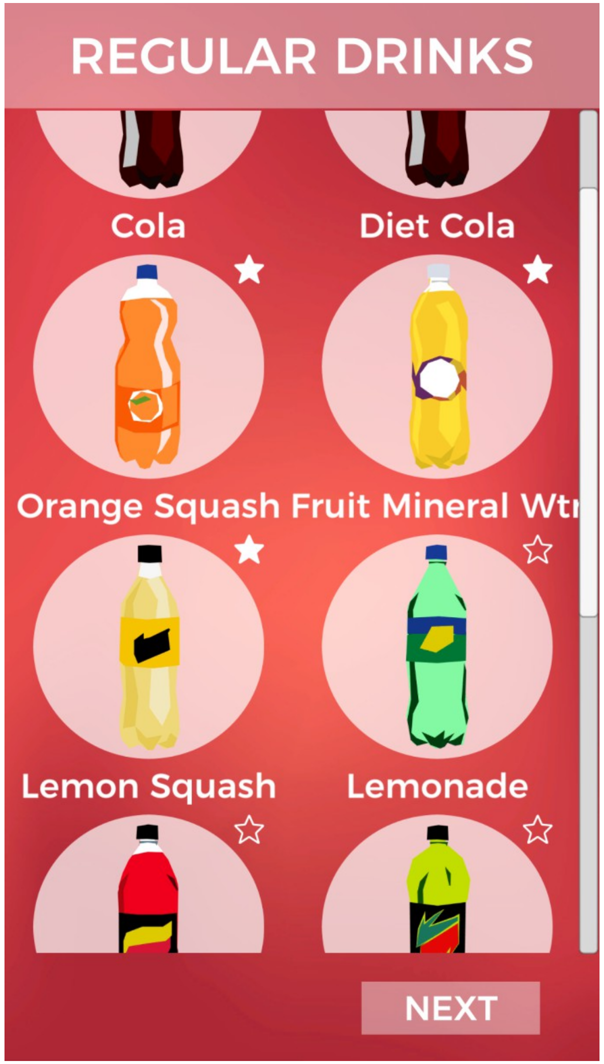

Multimedia Appendix 1

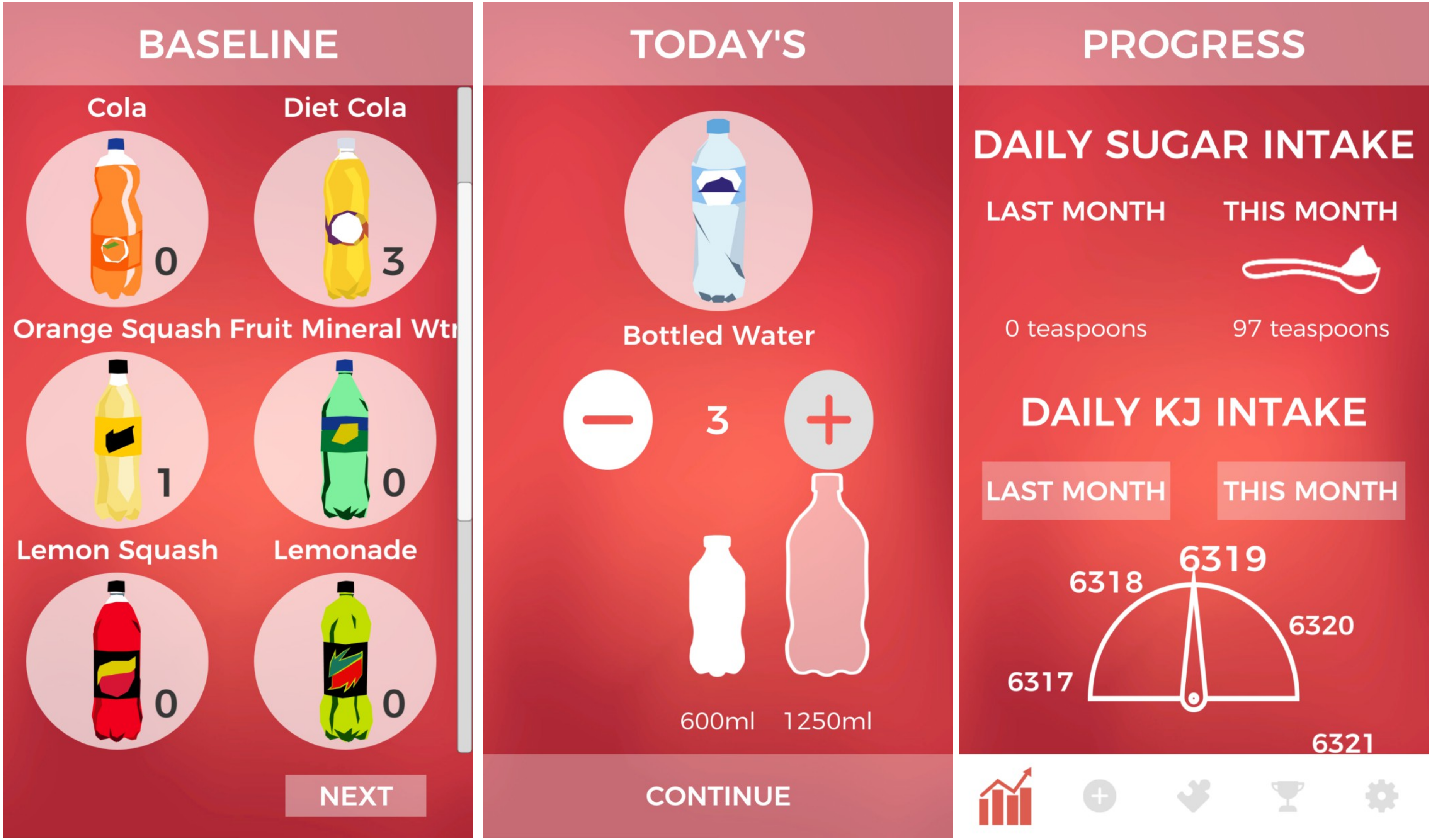

Multimedia Appendix 1

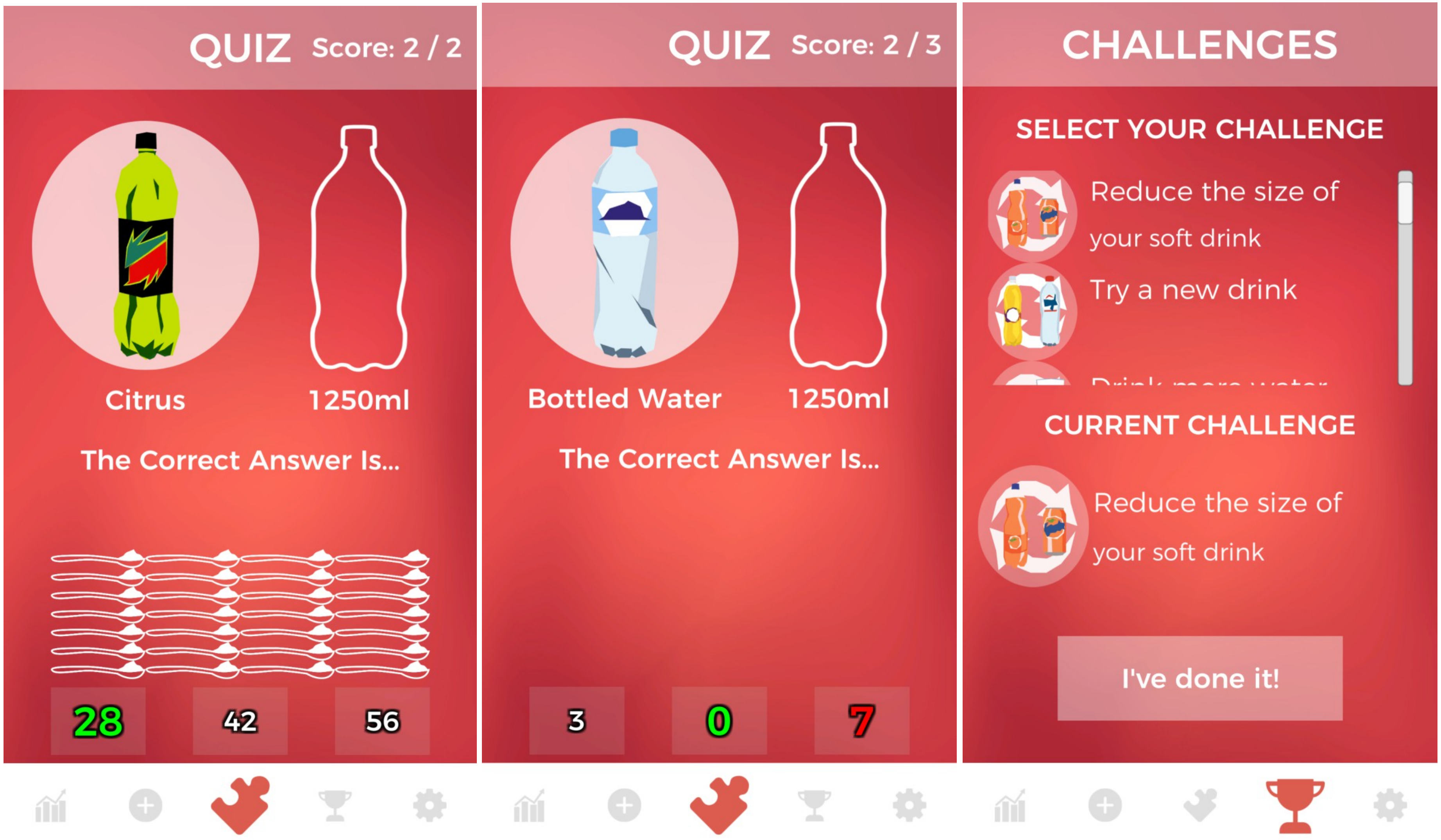

Supplement: Multimedia Appendix 1 [file mhealth_v5i12e192_app1.pdf]
