# Supplementary material for: A Smartphone App to Reduce Sugar-Sweetened Beverage Consumption Among Young Adults in Australian Remote Indigenous Communities: Design, Formative Evaluation and User-Testing
Source: JMIR Mhealth Uhealth. 2017 Dec 12;5(12):e192. doi: 10.2196/mhealth.8651 (PMC5743922; doi:10.2196/mhealth.8651)
Supplement: Multimedia Appendix 2 [file mhealth_v5i12e192_app2.pdf]

Interview Guide – eating behaviour and food attitudes

The reason for getting this information is because when we make the app, we want it to be as useful to you as possible. Talking to you now about these things will help to make sure that the way we make the app, and the information in it, is going to be the most useful to you and others. 'Drinks' means all drinks but alcohol.

1. What types of food and drinks make you feel strong and proud?

2. Do you worry at all about what you eat or drink?

3. Do you think that what you eat or drink affects your body or mind?

4. Are there things about what you eat or drink that you would like to change?
    a. What are these?
    b. And why would you like to change these?

5. Have you tried changing the way you eat or drink at all? (for example, following a diet, trying a new type of diet or cutting out some foods).
    a. How did you go? (what was your experience).
    b. Was there anything that you used to help you do this?
    c. Was there anything that you wished you could have used to help you but didn't have?
    d. Was there anything that made it especially hard?

6. Are there some drinks that you just love and would be really hard to give up?

7. If you were going to make changes to what you eat and drink, would you prefer to do it with your friends and family, or by yourself?

8. Are there things about food or drinks that you find interesting?

9. Are there things about food and drinks that you would like to know more about?

10. Who would you say has taught you the most about what is good to eat and drink and what is not good to eat and drink?
