# Supplementary material for: A Smartphone App to Reduce Sugar-Sweetened Beverage Consumption Among Young Adults in Australian Remote Indigenous Communities: Design, Formative Evaluation and User-Testing
Source: JMIR Mhealth Uhealth. 2017 Dec 12;5(12):e192. doi: 10.2196/mhealth.8651 (PMC5743922; doi:10.2196/mhealth.8651)
Supplement: Multimedia Appendix 3 [file mhealth_v5i12e192_app3.pdf]

Multimedia Appendix 3

Smartphone and app use questionnaire

1. What brand of smartphone do you have?
2. What IT devices do you own?
3. How long have you had your current smartphone?
4. What do you use you smartphone for?
5. How many times do you think you use your smartphone each day?
6. Do you often loan your phone to other people?
7. How often is your smartphone connected to the internet? Do you use Wi-Fi or mobile network in your community?
8. Do you use your local language when messaging friends and family on your phone and on social media?
9. Do you have a Facebook account?
10. Do you have the Facebook app on your phone?
11. Have you used social media or any apps to help you to change what you eat and drink?
12. Do you use other apps?
