# Supplementary material for: A Smartphone App to Reduce Sugar-Sweetened Beverage Consumption Among Young Adults in Australian Remote Indigenous Communities: Design, Formative Evaluation and User-Testing
Source: JMIR Mhealth Uhealth. 2017 Dec 12;5(12):e192. doi: 10.2196/mhealth.8651 (PMC5743922; doi:10.2196/mhealth.8651)
Supplement: Multimedia Appendix 4 [file mhealth_v5i12e192_app4.pdf]

Multimedia Appendix 3

Structured phone interview schedule for Stage 2 App testing

Modified System Usability Scale

1. Would you like to use the app more often?

   Yes No Maybe

   Comments

2. Did you find the app hard to use?

   Yes No Maybe

   Comments

3. Were there easy parts to using the app?

   Yes No Maybe

   Comments

4. Did you need help to use the app?

   Yes No Maybe

   Comments

5. Do you think the different screens worked well together?

   Yes No Maybe

   Comments

6. Do you think the app would be easy for anyone to use?

   Yes No Maybe

   Comments

7. Did the app take a lot of you time?

   Yes No Maybe

   Comments

8. Did you feel confident using the app?

   Yes No Maybe

   Comments

9. Do you feel you need to learn more information before using this app again?

   Yes No Maybe

Comments

Additional questions

10. Do you think you would you use the app?

Yes No Maybe

11. Did you have problems with any of the screens?

Yes No Maybe

12. Did you think the app looked good?

Yes No Maybe

12. What did you like **least** about the App?

1.

2.

3.

13. What did you like **best** about the App?

1.

2.

3.

14. Do you have any other comments about the App?

1.

2.

Multimedia Appendix 3

Thank you for your time.
